# Desferrioxamine Attenuates Pancreatic Injury after Major Hepatectomy under Vascular Control of the Liver: Experimental Study in Pigs

**DOI:** 10.1155/2012/714672

**Published:** 2012-06-25

**Authors:** Panagiotis Varsos, Constantinos Nastos, Nikolaos Papoutsidakis, Konstantinos Kalimeris, George Defterevos, Tzortzis Nomikos, Agathi Pafiti, George Fragulidis, Emmanuel Economou, Georgia Kostopanagiotou, Vassilios Smyrniotis, Nikolaos Arkadopoulos

**Affiliations:** ^1^Fourth Department of Surgery, School of Medicine, University of Athens, Attikon University Hospital, 1 Rimini Street, 12462 Athens, Greece; ^2^Second Department of Surgery, School of Medicine, University of Athens, Aretaieion University Hospital, 76 Vassilissis Sofias Avenue, 11528 Athens, Greece; ^3^Second Department of Anesthesiology, School of Medicine, University of Athens, Attikon University Hospital, 1 Rimini Street, 12462 Athens, Greece; ^4^Department of the Science Nutrition-Dietetics, Harokopio University, 70 Eleftheriou Venizelou Street, 17671 Athens, Greece; ^5^Department of Pathology, School of Medicine, University of Athens, Aretaieion University Hospital, 76 Vassilissis Sofias Avenue, 11528, Athens, Greece; ^6^Hormonal and Biochemical Laboratory, Aretaieion University Hospital, 76 Vassilissis Sofias Avenue, 11528 Athens, Greece

## Abstract

*Introduction*. Pancreatic injury can manifest after major hepatectomy under vascular control. The main mechanism involved seems to be remote oxidative injury due to “spillage” of reactive oxygen species and cytokines from the liver. The aim of this study is to evaluate the role of desferrioxamine in the prevention of pancreatic injury following major hepatectomy. *Methods*. Twelve Landrace pigs were subjected to a combination of major hepatectomy (70–75%), using the Pringle maneuver for 150 minutes, after constructing a porta-caval side-to-side anastomosis. The duration of reperfusion was 24 hours. Animals were randomly divided into a control group (*n* = 6) and a desferrioxamine group (DFX, *n* = 6). DFX animals were treated with continuous IV infusion of desferrioxamine 100 mg/kg. Pancreatic tissue injury, c-peptide and amylase concentrations, and pancreatic tissue oxidative markers were evaluated. *Results*. Desferrioxamine-treated animals showed decreased c-peptide levels, decreased acinar cell necrosis, and decreased tissue malondialdehyde levels 24 hours after reperfusion compared with the control group. There was no difference in portal pressure or serum amylase levels between the groups. *Conclusions*. Desferrioxamine seems to attenuate pancreatic injury after major hepatectomy under vascular control possibly by preventing and reversing production and circulation of oxidative products.

## 1. Introduction

Ischemia and reperfusion injury takes place during major hepatectomies due to the need for the use of vascular control techniques, as well as in liver transplantation and liver trauma. Although such maneuvers are invaluable in preventing excessive blood loss, they result in the production of cytokines and reactive oxygen species (ROS), which are responsible for induction of oxidative stress to the liver as well as to distant organs [1, 2]. Spillage of cytokines and inflammatory mediators has been shown to promote remote injury [3].

Hyperamylasemia and pancreatic injury has been well documented following major liver resections in patients with or without chronic liver disease [4, 5], as well as in experimental models [6]. Portal congestion, liver failure, and remote oxidative stress have been proposed as pathophysiologic mechanisms [3, 7]. Similar finding have been reported after liver transplantation [8, 9]. Although most often pancreatic injury can be subclinical, it can manifest as severe pancreatitis resulting in multiple organ failure, increasing morbidity and mortality following liver surgery [9, 10].

Desferrioxamine has been used in the past as an antioxidant in liver ischemia and reperfusion, as well as for the protection of remote organ injury after hepatectomy [11–13].

The aim of this study was to investigate the role of desferrioxamine as an antioxidant agent in the prevention of pancreatic injury that follows major hepatectomy under vascular control. 

## 2. Methods

This protocol was approved by the Animal Research Committee of the University of Athens and the Committee of Bioethics of Aretaieion Hospital. Care and handling of the animals was in accordance with European guidelines for ethical animal research. Twelve female Landrace pigs weighing 30–35 kg were used. The animals were randomly divided in two groups: a desferrioxamine treatment group (DFX, *n* = 6) and a control group (*n* = 6). DFX animals received a constant intravenous infusion of desferrioxamine beginning at the time of initiation of hepatic ischemia, until the end of the experiment. 

### 2.1. Surgical Procedure

All animals were subjected to major hepatectomy by removal of the left and median lobes, combined with ischemia of 150 minutes and a 24-hour reperfusion period, as described in the past [6]. A side-to-side portacaval anastomosis was performed using continuous 5-0 prolene sutures, in order to prevent splanchnic congestion. During the creation of the anastomosis, care was taken not to interrupt blood flow through the vessels. After the anastomosis, the left hepatic artery was ligated, and the hepatoduodenal ligament was clamped (Pringle maneuver). Afterwards, 70% hepatectomy was performed by resection of the median and left liver lobes. The liver remnant was kept ischemic for 150 minutes, and then the portacaval anastomosis was clamped, and portal blood flow was redirected back to the liver remnant by unclamping the hepatoduodenal ligament. A 20G catheter was then inserted in the portal vein through a side branch for portal pressure monitoring and portal blood sampling. The abdomen was closed, and the liver was reperfused for a 24-hour period during which the animals were kept under mechanical ventilation and monitored. Mean arterial pressure (MAP) and portal pressure (PP) were recorded, and blood samples were taken at the beginning of the reperfusion period and at 0, 6, 12, and 24 hours of reperfusion. At the end of the experiment, all animals were euthanized with intravenous infusion of thiopental 5 mg/kg and 2 g KCl, and pancreatic tissue was sampled for histological studies and measurement of malondialdehyde (MDA) and protein carbonyls content.

### 2.2. Desferrioxamine Administration Protocol

Desferrioxamine 100 mg/kg was administered continuously IV starting at the time of occlusion of the hepatoduodenal ligament (start of ischemia), until the end of the experiment. The total dose was divided in 66 mg/kg that was administered during the ischemic period until the 6th hour of reperfusion and 34 mg/kg that was administered after the 6th hour, until the end of the experiment. Animals in the control group received an equal volume of normal saline 0.9%. 

### 2.3. Tissue Oxidative Markers Content Assay

Blood samples were separated by centrifugation at 4000 rpm at 4°C for 20 minutes and stored at −80°C until analysis. Pancreatic tissue was placed to liquid nitrogen immediately after collection and then stored at −80°C until analysis. MDA content was determined in the membrane fraction of the tissue according to the protocol we have already described [6]. The total protein of the membrane fraction was determined by the Bradford method [14], and the MDA content was determined according to the method of Jentzsch et al. [15] using 100 *μ*g of membrane protein. Protein *carbonyls* were measured using the colorimetric assay kit from *Cayman Chemical* (Ann Arbor, MI). Results are expressed as nmoL per mg of tissue homogenate protein.

### 2.4. Histological Evaluation

Pancreatic tissues sampled were fixed in 4% formaldehyde, embedded in paraffin, and then cut into 3–5 *μ*m sections and stained with haematoxylin-eosin (HE) staining.

An expert pathologist then studied five sights of each sample in a blind manner. A modification of Schmidt et al. [16] grading was used in which the pancreatic injury was evaluated regarding congestion, pancreatic cell necrosis, inflammation, and hemorrhage and static necrosis according to Table 1.

### 2.5. Biochemical Analysis

After collection, blood samples were separated by centrifugation at 4000 rpm for 20 minutes, and amylase levels were determined in systemic circulation using the Dimension RXL system (Dade Behring, Dupond). c-peptide concentration was determined in portal circulation as a marker of endocrine pancreatic cell injury, using a porcine c-peptide RIA kit (Linco Research, Missouri, USA).

### 2.6. Statistical Analysis

Analysis of variance was used in order to determine statistical significance when the distribution was normal. When the distribution was not normal, when the data was ordinal, and when standard deviations differed significantly, the nonparametric Man-Whitney test was used. Normality was tested using the Kolmogorov-Smirnov technique. All calculations were carried out using SPSS 15.0 for Windows. The level of statistical significance was set to *P* < 0.05. Data are expressed as mean ± SD.

## 3. Results

### 3.1. Portal Pressure

There were no statistically significant differences in portal pressure between the two groups during the reperfusion period. In addition, there were no significant changes in portal pressure within groups during the reperfusion period.

#### 3.1.1. Pancreatic Histology

Pancreatic microscopy revealed pancreatitis in both groups. Necrosis, edema, hemorrhage, and leukocyte accumulation were present in both groups. Necrosis was significantly lower in the DFX group compared with control animals (2.7 ± 0.5 versus 1.5 ± 0.5, *P* < 0.05). No differences were noted in inflammatory infiltration, edema, and hemorrhagic foci. Although total pancreatitis score was lower in the DFX group, the difference failed to achieve statistical significance (*P* = 0.052), as shown in Table 2.

#### 3.1.2. Serological Markers of Pancreatic Injury

C-peptide levels followed an increasing pattern in both the groups during the first hours of reperfusion. In the control group, c-peptide was significantly higher compared to values immediately after reperfusion throughout the experiment (*P* < 0.05). In animals treated with desferrioxamine, c-peptide increased significantly after reperfusion until the 12-hour time point (*P* < 0.05). After 12 hours of reperfusion, c-peptide had a decreasing trend in the DFX group and did not have significant differences compared to values immediately after reperfusion. C-peptide values were significantly lower in the DFX group 24 hours after reperfusion compared with the control group (*P* = 0.037), as shown in Table 3.

Serum amylase levels increased during reperfusion in both groups until 12 hours after reperfusion, but the increase was not statistically significant. At 24 hours, amylase levels continued to increase in control group, still with no statistical significance. No significant differences between groups were observed in any time point, as shown in Table 4.

#### 3.1.3. Pancreatic Tissue Protein Carbonyls and MDA Content

Pancreatic MDA content was decreased in the DFX group compared with the control group 24 hours after reperfusion (*P* = 0.005). In the contrary, there was no difference in pancreatic protein carbonyls content as shown in Table 5. 

## 4. Discussion

Ischemia and reperfusion injury that occurs during liver transplantation, liver resections under vascular control, and liver trauma surgery has an impact on the liver as well as remote organs [6, 8, 11, 13, 17, 18]. The spillage of cytokines and reactive oxygen species have been implicated in the pathogenesis of remote organ injury [3, 6, 11, 13, 17, 19–21]. Pancreatic injury has been reported following liver ischemia reperfusion in liver transplantation and in major liver resections [18, 20, 21]. 

Antioxidants have been widely used to prevent or reverse reperfusion injury of the liver [22]. Desferrioxamine has been commonly used in ischemia and reperfusion injury [23–25] and has been shown to attenuate ischemic and oxidative injuries to the liver and other tissues [12, 26–33], as well as remote injury to the intestinal mucosa, the lung, and the myocardium [11, 13, 17]. Desferrioxamine chelates iron, preventing the production of oxygen free radicals though the Fenton equation, and induces the expression of hypoxia-inducible factor 1-alpha (HIF-1alpha) giving protection against hypoxic states [34, 35]. Desferrioxamine blocks an alternate pathway (Fenton reaction) in the production of reactive oxygen species compared to other antioxidants. Desferrioxamine has also been shown to have antioxidative properties by means of scavenging free radicals [36]. Recently, desferrioxamine was used for the prevention of pancreatitis induced by liver transplantation in rats [18].

We have already shown that the pathophysiology of pancreatitis following major hepatectomy under vascular control is multifactorial [6]. It involves portal hypertension during the Pringle maneuver leading to direct congestion of the pancreas [7], postoperative portal hypertension due to the decreased intrahepatic portal vasculature following massive resections [37–40], spillage of cytokines, oxidative substances, and reactive oxygen species which produce tissue injury [3, 20]. 

We have used an already described experimental model of major hepatectomy under vascular control without intraoperative portal hypertension during the Pringle maneuver [41]. Our data show that treatment with desferrioxamine attenuates pancreatic tissue necrosis compared with the control group 24 hours after reperfusion, while there is no difference in tissue edema, hemorrhage, and inflammatory infiltration. In both groups, interlobular and interacinar edema was noticed, while tissue was disorganized, showing necrosis of acinar cells, infiltration of a few leukocytes, and interstitial hemorrhage. Li et al. have reported similar findings after liver transplantation in rats. They reported attenuation of acinar cell necrosis and decreased edema 6 hours after liver transplantation in animals treated with desferrioxamine. This effect was even more remarkable 24 hours after transplantation [18]. 

According to the histological findings, serum markers of pancreatic cell injury were increased during the reperfusion period. C-peptide has been used in the past as a marker of endocrine cell damage, as it has been shown to correlate with morphological changes to the pancreas during injury [6, 42]. C-peptide was significantly lower in animals treated with desferrioxamine at the end of the experiment, while differences in amylase levels failed to achieve statistical significance in the same group. We have already shown that pancreatic injury takes place early in the postoperative period, and that portal MDA content increases during the first hours of the reperfusion period after hepatectomy under vascular control. This effect peaks at 12 hours postoperatively and afterwards starts to disappear, as the percentage increase of portal MDA content in our previous study was higher in the 12-hour reperfusion time point compared to 24 hours [43]. This pattern is in accordance with the early phase of the ischemia reperfusion injury that has been documented in the literature [44, 45]. We attributed the sudden increase of c-peptide at 12 hours in both groups to oxidative injury to the pancreas that peaked at 12 hours postoperatively as demonstrated in our previous work.

In our study, we evaluated pancreatic tissue protein carbonyls and malondialdehyde (MDA) as markers of lipid peroxidation and oxidative injury. Pancreatic MDA was significantly lower following desferrioxamine treatment 24 hours after reperfusion. However, there was no difference in the levels of pancreatic tissue protein carbonyls. This could be explained by the fact that these biochemical processes are modulated by different mechanisms in this organ. Alexandris et al. have reported that lipid and protein oxidation can have different kinetics, resulting in different recovery times, thus influencing tissue levels [46].

In conclusion, our study supplies evidence that desferrioxamine attenuates pancreatic injury after major hepatectomy under vascular control. Desferrioxamine can decrease the production and systemic “spillage” of inflammatory and toxic mediators (oxidative products), that are produced during liver oxidative injury. The protective mechanism of desferrioxamine seems to be a combination of (1) chelation of redox active iron and prevention of oxygen free radicals production in the liver during reperfusion, thus preventing the production of inflammatory mediators and their circulation; (2) scavenging of reactive oxygen species produced in the liver and in other organs; (3) binding of redox active iron and prevention of its release from the liver during reperfusion.

## Figures and Tables

**Table 1:** Pancreatitis histological grading.

*Edema*	
No edema present	0
Interlobular edema	1
Interacinar edema	2
Intercellular edema	3
Acinar cell necrosis	
No necrotic cells	0
1–4 necrotic cells/field	1
5–10 necrotic cells/field	2
>10 necrotic cells/field	3
Inflammation	
No leukocytes	0
1–4 leukocytes/field	1
5–10 leukocytes/field	2
>10 leukocytes/field	3
Hemorrhage and fat necrosis	
Absent	0
1–3 foci	1
3–5 foci	2
>5 foci	3

**Table 2:** Pancreatic tissue injury scoring.

	Group	Mean	Std. deviation	*P* value compared to control group
Total score	Control	9.5	1.4	0.052
	DFX	7.7	1.5	
Edema	Control	2.2	0.4	0.34
	DFX	2.0	0	
Necrosis	Control	2.7	0.5	**0.004**
	DFX	1.5	0.5	
Inflammation	Control	2.5	0.5	0.11
	DFX	1.8	0.8	
Hemorrhage	Control	2.2	0.8	0.66
	DFX	2.3	0.5	

**Table 3:** C-peptide levels (ng/mL) in portal circulation during the reperfusion period.

Timepoint	Group	Mean c-peptide levels in portal circulation (ng/mL)	Std. deviation	*P* value compared to the same time point of the control group
Baseline	Control	0.42	0.17	0.98
	DFX	0.42	0.22	
0 hours of reperfusion	Control	0.71	0.29	0.70
	DFX	0.78	0.34	
6 hours of reperfusion	Control	0.97^†^	0.30	0.25
	DFX	1.20^‡^	0.34	
12 hours of reperfusion	Control	2.02^†^	1.34	0.15
	DFX	1.27^‡^	0.35	
18 hours of reperfusion	Control	1.56^†^	0.67	0.08
	DFX	0.95	0.39	
24 hours of reperfusion	Control	1.62^†^	0.78	**0.037**
	DFX	0.90	0.26	

^†^ *P* < 0.05 compared to c-peptide values on the control group immediately after reperfusion.

^‡†^ *P* < 0.05 compared to c-peptide values on the DFX group immediately after reperfusion.

**Table 4:** Serum amylase levels (U/mL) in systemic circulation during the reperfusion period.

Timepoint	Group	Mean serum amylase concentration (U/mL)	Std. deviation	*P* value compared to the same time point of the control group
0 hours after reperfusion	Control	1201	244	0.65
	DFX	1300	465	
6 hours after reperfusion	Control	1555	584	0.91
	DFX	1599	859	
12 hours after reperfusion	Control	1732	474	0.88
	DFX	1686	576	
24 hours after reperfusion	Control	1828	508	0.055
	DFX	1252	138	

**Table 5:** Pancreatic tissue protein carbonyls and malondialdehyde (MDA) levels (nmoL/mg protein) 24 hours after reperfusion.

	Group	Mean value	Std. deviation	*P* value compared to the same time point of the control group
Tissue protein carbonyls (nmoL/mg protein)	Control	3.2	1.8	0.15
	DFX	4.8	3.9	
Tissue MDA (nmoL/mg protein)	Control	2.4	0.8	** 0.005**
	DFX	1.1	0.1	
